# Supplementary material for: The Evolution and Characterization of the RNA Interference Pathways in Lophotrochozoa
Source: Genome Biol Evol. 2024 May 7;16(5):evae098. doi: 10.1093/gbe/evae098 (PMC11114477; doi:10.1093/gbe/evae098)
Supplement: evae098_Supplementary_Data [file evae098_supplementary_data.zip › Supplementary_materials.zip!/selected_miRNAs/NoGe_selected_miRNAs/known_miRNA_families_pdf_files/NMRB01000201.1_Notospermus_geniculatus_strain_Ushimado_scaffold201,_whole_genome_shotgun_sequence_7104.pdf]

Provisional ID	:	NMRB01000201.1_Notospermus_geniculatus_strain_Ushimado_scaffold201,_whole_genome_shotgun_sequence_7104
Score total	:	9774.9
Score for star read(s)	:	3.9
Score for read counts	:	9766.3
Score for mfe	:	1.7
Score for randfold	:	0
Score for cons. seed	:	3
Total read count	:	19168
Mature read count	:	19081
Loop read count	:	0
Star read count	:	87

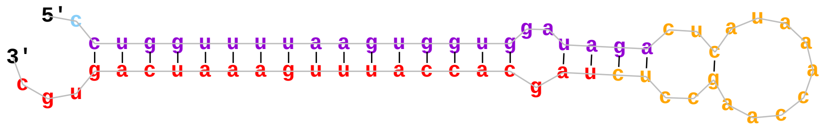

Genome coordinates: NMRB01000201.1 525434-525495

pre-miRNA sequence:
CUGGUUUUAAGUGGUGGAUAGACUGGUUUUAAGUGGUGGAUAGACUCAUAAACCAAGCCUCUAGCACCAUUUGAAAUCAGUGC

Mature sequence: UAGCACCAUUUGAAAUCAGUGC

Aligned mature sequence of the *N. geniculatus* pre-miRNA with the top 5 hits from the BLAST search against the pre-miRNA mirGeneDB set:

```
                            **************** **
nge_Mir-29-P1l              uagcaccauuugaaaucagugc
Xla-Mir-29-P2b4_3p          uagcaccauuugaaaucgguua
Xla-Mir-29-P2d3_3p          uagcaccauuugaaaucgguua
Xla-Mir-29-P2d4_3p          uagcaccauuugaaaucgguua
Xtr-Mir-29-P2b_3p           uagcaccauuugaaaucgguua
Xtr-Mir-29-P2d_3p           uagcaccauuugaaaucgguua
```
